# Short- and Middle-Term Nephroprotective and Cardioprotective Effects of Pentoxifylline in Patients with Diabetic Nephropathy: A Randomized Controlled Trial

**DOI:** 10.3390/medsci14010026

**Published:** 2026-01-06

**Authors:** Oliva Mejía-Rodríguez, Marcela Ávila-Díaz, Carmen Prado-Uribe, María Eugenia Galván-Plata, Helios Vega Gómez, Cleto Álvarez-Aguilar, Miguel Angel Cuevas-Budhart, Ramón Paniagua

**Affiliations:** 1 Centro de Investigación Biomédica de Michoacán, Instituto Mexicano del Seguro Social, Morelia C.P. 58341, Michoacán, Mexico; olivamejia@yahoo.com; 2 Coordinación de Investigación en Salud, Instituto Mexicano del Seguro Social, Avenida Cuauhtémoc 330, Col. Doctores, Ciudad de Mexico C.P. 06725, Mexicoangel_budhart@hotmail.com (M.A.C.-B.); 3 Hospital General Regional 1, Instituto Mexicano del Seguro Social, Morelia C.P. 58341, Michoacán, Mexico; 4 Coordinación Auxiliar Médica de Investigación en Salud, Instituto Mexicano del Seguro Social, Morelia C.P. 58341, Michoacán, Mexico; 5 Facultad de Ciencias Médicas y Biológicas “Dr. Ignacio Chávez”, Universidad Michoacana de San Nicolas de Hidalgo, Morelia 58020, Michoacán, Mexico

**Keywords:** Chronic Kidney Disease, Chronic Kidney Disease progression, Type 2 Diabetes mellitus, Pentoxifylline, cardioprotection, nephroprotection

## Abstract

**Background/Objectives**. Pentoxifylline (PTF) is an effective treatment to delay the progress of Diabetic Nephropathy; it also has beneficial effects on heart failure, two closely related clinical conditions. However, the simultaneous Nephroprotective and Cardioprotective effects of PTF have not been appropriately analyzed. The objective of this study was to analyze if both effects occur together in Diabetic patients. **Material and Methods**. A Randomized Controlled Trial was performed to compare Placebo (P-G) vs. PTF (400 mg/8 h) (PTF-G) in patients with CKD stages III–IV (KDIGO) due to Diabetic Nephropathy. Creatinine-Cystatin C-based Estimated Glomerular Filtration Rate (eGFRCysCCr) and Microalbuminuria were evaluated at baseline, six, and twelve months. Echocardiographic assessment of heart morphology and function was performed. **Results**. Ninety-three patients were available for the final analysis, 52 in the PTF group and 41 in the P group. There were no differences in clinical and biochemical parameters between groups at baseline. At 6 months, microalbuminuria changed 27.96 ± 43.06 in P-G and −30.27 ± 41.48 mg/24 h in PTF-G (*p* < 0.001), eGFRCysCCr changed −1.55 ± 7.10 in P-G and 1.40 ± 7.28 mL/min/1.73 m^2^ in PTF-G (*p* = 0.083), and left ventricular mass index (LVMI) changed 10.86 ± 16.40 in P-G and −2.71 ± 19.52 g/m^2^ in PTF-G (*p* = 0.001). At 12 months, microalbuminuria changed 24.10 ± 43.28 in P-G and −43.18 ± 52.13 mg/24 h in PTF-G (*p* < 0.001), eGFRCysCCr changed −6.55 ± 10.18 in P-G and 0.98 ± 8.17 mL/min/1.73 m^2^ in PTF-G (*p* = 0.008), and LVMI changed 20.79 ± 20.06 in P-G and −0.82 ± 25.95 g/m^2^ in PTF-G (*p* = 0.028). No significant adverse events occurred in any group. **Conclusions**. Pentoxifylline is a safe and effective treatment with combined Nephroprotective and Cardioprotective effects in advanced diabetic nephropathy.

## 1. Introduction

Chronic Kidney Disease (CKD) is recognized as a global challenge for health systems due to the magnitude of human, infrastructural, and economic resources it demands. CKD affects more than 10% of the general population which accounts for over 850 million people worldwide [1]. On the other hand, Type 2 Diabetes mellitus (T2DM) is a significant associated challenge because it is the most frequent cause of CKD. It has been estimated that there are 589 million people between 20 and 79 years old who have Diabetes, which accounts for 11.1% of the population in that age group. It is further estimated that this number will grow to 853 million (13%) by 2050 [2]. This epidemiological trend represents a growing public health burden, particularly in middle-income countries, where limited resources amplify the social and economic impact of diabetic kidney disease.

Most clinical practice guidelines for CKD identify risk factors for CKD progression and include strategies to counteract them. Lifestyle changes such as smoking, a sedentary lifestyle, and, very significantly, a diet with restricted sodium and protein intake, primarily from animal sources, are prominent. Pharmacological measures emphasize the blockade of the renin–angiotensin–aldosterone system and, more recently, the use of sodium-glucose cotransporter 2 (SGLT2) inhibitors [3]. However, despite these advances, progressive renal decline and cardiovascular complications continue to be observed in a considerable number of patients, which highlights the need for additional, affordable, and complementary therapeutic strategies.

Pentoxifylline (PTF), a molecule from the methylxanthine family, has been used since 1980 [4] in the management of peripheral vascular insufficiency. PTF decreases blood viscosity, increases erythrocyte deformability, inhibits erythrocyte and platelet aggregation, stimulates fibrinolysis, and reduces plasma fibrinogen and tumor necrosis factor-alpha (TNF-α) levels. In connective tissue, it increases collagenase activity and decreases collagen, fibronectin, and glucosamine, while also decreasing the generation of free radicals [5,6,7,8,9,10].

In early clinical studies, PTF was shown to reduce albuminuria in both type 1 diabetes mellitus (T1DM) and T2DM patients, as well as in two subgroups, those with microalbuminuria and those with macroalbuminuria. PTF decreased significantly plasma levels of TNF-α, suggesting anti-inflammatory and endothelial-protective effects [5,6]. In the PREDIAN trial, it was demonstrated that PTF slowed the decline in glomerular filtration rate and reduced urinary albumin excretion when it was added to standard therapy [7,9]. Experimental studies in animal models confirmed these findings, showing reductions in oxidative stress and inflammation markers such as nitric oxide, advanced oxidation protein products, and TNF-α, while increased thiols concentration [8].

Based on these properties, it has been used to slow the progression of CKD. Its usefulness has been highlighted in several meta-analyses, where high-sensitivity C-reactive protein (hsCRP) levels were improved, urinary albumin excretion rates were reduced, the improvement in eGFR from baseline was increased, and fasting plasma glucose (FPG) levels were decreased in the PTF treatment group. However, the small sample size considered, the different therapy dosages used, and the low methodological quality led to poor precision of estimates, and this has also been pointed out [11,12,13,14,15,16,17]. PTF has also been used successfully in the management of atherosclerosis and endothelial dysfunction [18,19] and has been considered for chronic heart failure [19]; however, in patients with CKD, clinical or subclinical CHF is a frequent comorbidity [19,20], and the usefulness of PTF simultaneously to protect from both entities has not been sufficiently studied. The objective of this study was to analyze the short- and medium-term renoprotective and cardioprotective effects of PTF in patients with CKD due to diabetic nephropathy.

## 2. Materials and Methods

Design. A double-blinded, randomized, controlled clinical trial (RCT) was conducted in as per-protocol design. The first patient was enrolled in May 2018, and recruitment continued until July 2020. All participants were followed for twelve months after enrollment, with final assessments completed in June 2021.

Patients. Patients with T2DM and advanced CKD treated at the Mexican Social Security Institute (IMSS) were included. Eligible participants were aged between 18 and 70 years, with creatinine clearance ≤60 mL/min, proteinuria or microalbuminuria, and of either sex. Patients with infections, metabolic imbalance, or acute complications within the previous 30 days were excluded. Additional exclusion criteria included uncontrolled hypertension, clinical symptoms of heart diseases, psychiatric disorders, use of immunosuppressants, or herbal medicine, seropositivity for HIV, hepatitis B, or C. Screening for metabolic imbalance and acute complications was conducted through clinical evaluation, review of recent laboratory tests, and confirmation by the attending physician at enrollment.

Patients were invited to participate by their attending physician, who, along with a trained nurse in clinical research, provided them with information about the protocol and obtained their informed consent. The project was approved by the institution’s National Research and Ethics Committees (approved R-2015-785-065, approval date: 29 August 2019 and registered at ClinicalTrials.gov, NCT03664414, 7 February 2019). It was also approved by Local Hospital Research and Ethics Committees.

Intervention. Participants were randomly assigned, through a computer-generated sequence, to one of two treatment groups. The Pentoxifylline group (PTF-G) received 1200 mg per day, administered orally in three divided doses, whereas the Placebo group (P-G) received identical tablets containing an inert compound every eight hours. PTF dose were scaled from half of the recommended dose to the complete dose in a week.

Randomization was conducted by an independent pharmacist with no involvement in patient care, data collection, or analysis. The study medications were identical in size, shape, and color, ensuring full blinding of participants, investigators, and outcome assessors. Both groups continued receiving standard therapy according to guideline-recommended doses for the management of CKD. Standard therapy was used in nearly all participants, with 92% of patients in the PTF-G and 90% in the P-G receiving Angiotensin-Converting Enzyme Inhibitors (ACEIs) or Angiotensin Receptor Blockers (ARBs) at baseline. SGLT2 inhibitors, GLP-1 agonists, or non-steroidal MRAs were not available in the institution at the time of study.

Primary end points. Differences between groups were assessed in the following variables: microalbuminuria, estimated glomerular filtration rate derived from serum creatinine and cystatin C (eGFRCysCCr), left ventricular mass index (LVMI), and serum levels of NT-proBNP.

Microalbuminuria was selected as the primary endpoint used for sample size calculation due to its sensitivity to short-term therapeutic changes. eGFRCysCCr and LVMI were prespecified as co-primary functional and structural outcomes.

Secondary end points. Secondary analyses included evaluation of metabolic control (HbA1c, lipids, and blood pressure), inflammatory biomarkers, body composition, and the incidence of adverse events during follow-up.

Data Collection. Demographic and clinical variables were collected from the clinical record and through interviews with the attending physician and a nurse trained in clinical trial management. Patients were seen monthly for clinical evaluation and assessment of treatment adherence through specific questions and tablet counting.

Follow-up. Biochemical, body composition, and echocardiographic assessments were performed at baseline and every six months. Biochemical tests included: blood count, glucose, cholesterol, triglycerides, uric acid, HDL-C, LDL-C, glycated hemoglobin, SGOT, SGPT, serum albumin, and serum electrolytes (Na, Cl, K) were assessed using standard methods.

Body composition. Total body water (TBW), intracellular water (ICW), extracellular water (ECW), lean body mass (LBM), and fat mass (FMM) were quantified by multifrequency bioimpedance spectroscopy (Fresenius BCM^®^, Bad Homburg, Germany), with each measurement performed under fasting conditions and after voiding blader to minimize variability.

Echocardiogram. Echocardiographic evaluation (Phillips Medical System, Model iEEE33, Andover, MA, USA) was performed according to the recommendations of the American Society of Echocardiography [21]. Left ventricular mass (LVM) was calculated using the Deveraux formula; left ventricular hypertrophy was defined as a LVMI equal to or greater than 95 g/m^2^ in women and 115 g/m^2^ in men [22]. Body surface area was calculated using the Masteller formula [23]. Diastolic function was assessed following the recommendations of the American Society of Echocardiography, which considers diastolic dysfunction if it meets three or more of the following criteria: e/e ratio > 14 m, septal velocity < 7 cm/s or lateral velocity < 10 cm/s, tricuspid regurgitation > 2.8 m/s, left atrial volume index > 34 mL/m^2^ b.s. [24].

Statistical Analysis. Categorical variables were expressed as frequencies and percentages, and continuous variables as mean ± standard deviation. Between-group differences were assessed using the binomial, chi-square test or Student’s *t* test, as appropriate. Temporal and inter-group changes from baseline were evaluated with repeated-measures ANOVA. Assumptions for ANOVA, including normality and sphericity, were verified, and Greenhouse–Geisser corrections applied when required.

The sample size was calculated using microalbuminuria as the primary endpoint for power estimation. Based on a previous study analyzing the PTF effect on albuminuria and microalbuminuria in T1DM and T2DM Mexican patients with similar demographic and lifestyle characteristics, where reductions of at least 50% were seen [6], the sample size was estimated to be 40 patients per group (α = 0.05 and β = 0.20) [25]. A post hoc verification of statistical power was performed using the final sample size and observed results. The study was powered exclusively to detect changes in microalbuminuria. Analyses of eGFRCysCCr and LVMI were exploratory and not formally powered for hypothesis testing.

The primary analysis followed a per-protocol approach, with missing data handled by case-wise exclusion. All analyses were performed using SPSS v25 (IBM Corp., Armonk, NY, USA), and a *p* value < 0.05 was considered statistically significant.

Adherence and Safety Monitoring. The research team conducted clinical follow-up at each visit to assess adherence and monitor safety by self-reported complaint and count of pills consumed.

## 3. Results

**Figure 1:**
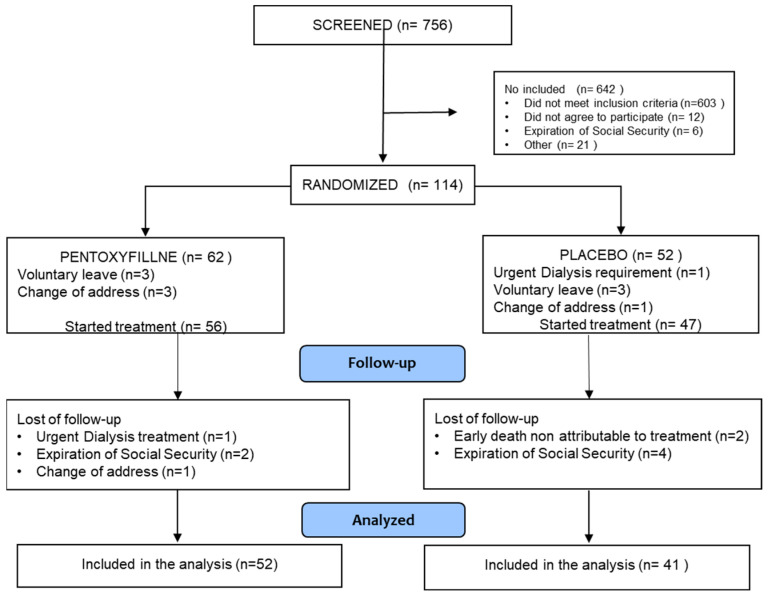
The Figure shows the patients flow throughout the study.

The screening, selection, and follow-up process is summarized in Figure 1. A total of 114 patients were assessed for eligibility; 62 were randomized to the PTF-G and 52 to the P-G. Patient dropout rates from randomization to the start of treatment were compared between groups (P-G: 5 patients, 9.62%; PTF-G: 6 patients, 9.68%; *p* = 0.52). Dropout rates from the start of treatment to the end of the study were also compared (P-G: 6 patients, 12.75%; PTF-G: 4 patients, 7.14%; *p* = 0.551). There was no difference between groups.

Baseline characteristics are summarized in Table 1. No significant differences were observed between the groups in demographic, clinical, body composition, biochemical, renal function, or echocardiographic variables, confirming adequate randomization. Both groups showed similar age distribution, sex proportion, and metabolic control parameters at baseline.

**Figure 2:**
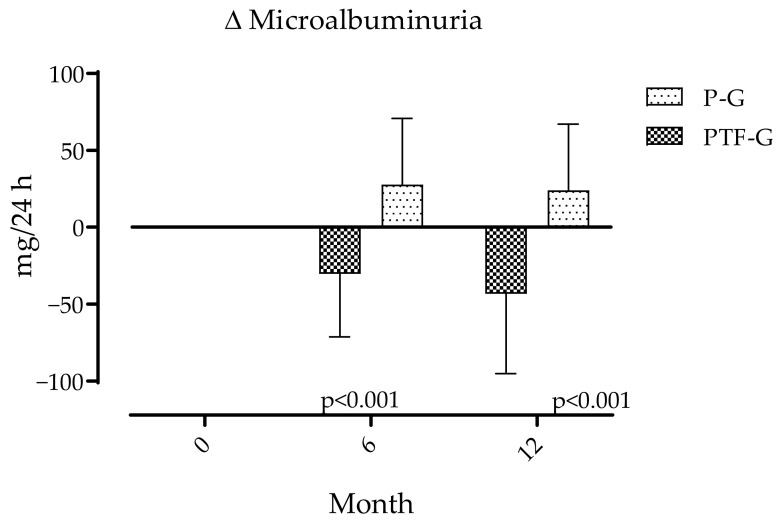
Changes in microalbuminuria over 12 months. Values increased in the placebo group and decreased in the pentoxifylline group. Data are expressed as mean ± SD. Between groups and temporal and interaction effects were assessed using repeated-measures ANOVA with Greenhouse–Geisser correction when required. There was significant time-group interaction (*p* < 0.01).

**Figure 3:**
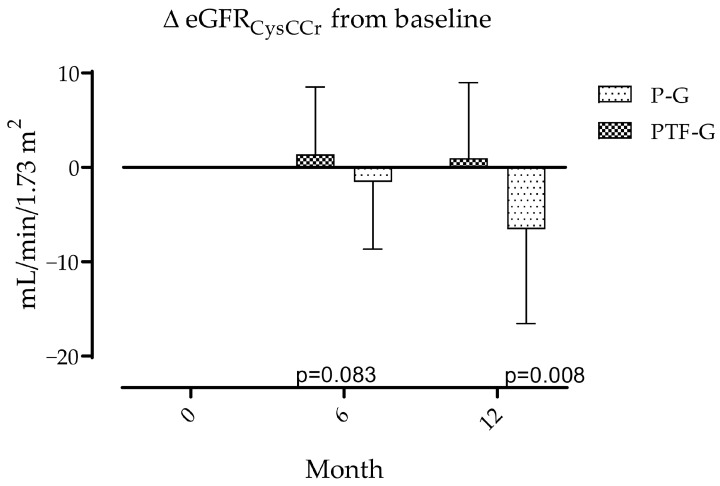
Changes in eGFRCysCCr over 12 months. Values declined in the placebo group and remained stable in the pentoxifylline group, with a significant difference at 12 months. Data are expressed as mean ± SD. Between-group, temporal and interaction effects were evaluated using repeated-measures ANOVA with Greenhouse–Geisser correction when required. Interaction group-time was significant at 12 months only.

Primary outcomes. Effects of PTF on Albuminuria are expressed as changes from baseline values (Table 1) to months 6 and 12. Microalbuminuria showed divergent patterns between groups. It tended to increase in P-G and decrease in PTF-G. Differences were significant at 6 and 12 months are shown in Figure 2. Over the same periods, eGFR calculated from the combination of values of creatinine and cystatin C shows more pronounced decrements from baseline values (Table 1) in the P-G than in the PTF-G at both 6 and 12 months, but only differences at 12 months were statistically significant (Figure 3).

**Figure 4:**
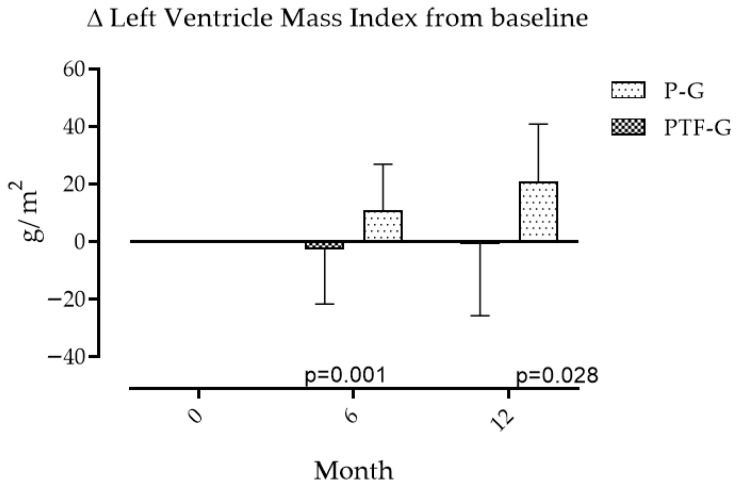
Changes in left ventricular mass index (LVMI) over 12 months. LVMI increased in the placebo group and decreased in the pentoxifylline group. Data are expressed as mean ± SD. Group, time and interaction effects were assessed using repeated-measures ANOVA with Greenhouse–Geisser correction when required. Interaction group-time was significant at 12 months only.

The LVMI remained without changes from baseline (Table 1) at 6 and 12 months in the PTF-G and increased in the P-G with statistically significant differences between groups (Figure 4) in the same periods. No differences were found between the groups in NT-proBNP concentrations at any of the periods evaluated. However, changes in NT-pro-BNP levels correlate with changes in eGFR_CysCCr_ (r = 0.626, *p* > 0.01), but not with changes in LVMI (r = 0.016, *p* = 0.906).

The power of the sample size was estimated for the primary outcomes to support the results. For final changes in microalbuminuria, the power was 0.91, and for changes in LVMI, it was 0.8, which are within standard limits. When the effect of dropouts was included in the analysis, no change in significance was observed.

No significant differences from baseline values (Table 1) were found across the evaluation periods in other variables, including weight, blood pressure, metabolic control, or inflammatory markers, as it is shown in Table 2. There was a trend to reduction in ECW volume, but not in other indicators of body composition.

Adherence and Safety. Adherence remained above 95% in both groups, and no treatment withdrawals occurred. No serious adverse events (such as deaths, needs of dialysis, cardiovascular events, infections, or hyper or hypoglycemia) were seen. Only mild gastrointestinal symptoms were reported during the first week of treatment with no difference between groups.

## 4. Discussion

The main findings of this study were the short- and medium-term effects of PTF treatment on reducing albuminuria, attenuating the decline in eGFR, and preventing an increase in the LVMI. The findings are in line with the nephroprotective and cardioprotective effects observed in T2DM and non-renal populations, respectively.

Recent literature has noted that adjunctive therapies for diabetic kidney disease often yield heterogeneous renal and cardiac responses, and that few studies assess both domains simultaneously. Within this context, the consistent changes observed in Albuminuria, eGFRCysCCr, and LVMI in our trial provide synchronized renal and cardiac information under the same randomized design, contributing to an area where evidence remains limited. This integrated perspective helps contextualize our findings within prior work evaluating together the reno- and cardioprotective potential of pentoxifylline [26].

The usefulness of PTF in reducing albuminuria has been demonstrated since the 1980s [5] and has been consistently documented in various meta-analyses published in the last fifteen years [11,12,13,14,15,16,17]. However, it is important to note the heterogeneity of the comparisons. These have been against placebo or against conventional treatments, including drugs that act on the renin–angiotensin–aldosterone system or against molecules with actions on glycemic control, endothelin, or the development of fibrosis [11,12,13,14,15,16,17].

Despite its usefulness, PTF is not among the treatments recommended in Clinical Practice Guidelines for the management of CKD or CKD secondary to diabetic nephropathy. This is probably due to the heterogeneity of clinical studies and the broad spectrum of PTF effects, which range from hemorheological effects to anti-inflammatory effects, attenuation of oxidative stress, and the management of heart failure.

This dual mechanism is clinically relevant given the well-documented bidirectional relationship between kidney and heart failure. PTF reduces the progression of atherosclerosis [18] and endothelial damage in diabetic patients with CKD [19]; however, the simultaneous effect of PTF on the kidney and heart has not been sufficiently explored. In an analysis of the reno- and cardioprotective effects of several drugs, PTF was not found among the most prominent, being surpassed by sodium-glucose cotransporter-2 inhibitors and aldosterone receptor agonists [20]. In another meta-analysis, it has been found to be useful in the treatment of heart failure [27] and potentially useful in other cardiovascular disorders [28]. The immunomodulatory effect has been pointed out as the mechanism involved [29].

In a post hoc analysis, performed seven years after the finish of the original study, with 24 patients in the control group and 11 in the PTF group, it was found that PTF delayed the need for dialysis and significantly reduced cardiovascular mortality [30]. The effect was independent of diabetes mellitus and was more pronounced in albuminuric patients. In a prospective national cohort study in Taiwan designed to evaluate the effect of PTF plus ACEIs vs. PTF plus ACEIs/ARBs in patients with CKD stage 5 without dialysis, after propensity score-matching, PTF was associated with a lower risk of dialysis or death in ACEI/ARB users or ARB users [31]. In a previous retrospective study with a median follow-up period of 2.5 years, in which PTF was administered in addition to ACEIs or ARBs, patients under PTF treatment had a better nephroprotective effect, but neither all-cause death nor cardiovascular death differed from patients without PTF [32]. Even though controversial, PTF reduced all-cause cardiovascular death and/or cardiovascular death. None of the aforementioned studies reported echocardiographic data. In non-renal patients with idiopathic dilated cardiomyopathy, PTF reduced levels of inflammatory cytokines and improved left ventricular performance [33,34]. Positive cardiovascular effects of PTF have also been found in experimental studies. It reduced inflammation, reduced fibrosis, and cardiac remodelation [35,36].

Our findings corroborate PTF’s nephroprotective properties and demonstrate its capacity to prevent cardiac remodeling, as reflected by the stability of LVMI. Even as a surrogate structural marker of major cardiovascular events, the finding is significant since it provides additional information supporting previous findings on the reduction in mortality, specifically cardiovascular mortality, with PTF treatment. Interestingly, circulating NT-proBNP levels were not significantly modified, a result that may seem paradoxical. However, correlation analysis revealed that NT-proBNP variation was primarily influenced by changes in eGFR rather than by LVMI, suggesting that renal function exerts a more substantial effect on this biomarker in advanced CKD.

As in other studies, we found no effect of PTF on blood pressure [37] or metabolic control [13]. The reduction in extracellular volume is likely related to the improvement or preservation of renal function.

The search for effective therapies for advanced diabetic complications remains a clinical priority. Pentoxifylline continues to represent a viable alternative, either as combination therapy with standard treatments or as an adjunctive agent when other regimens are insufficient [13,16,38]. Its low cost, safety, and availability may offer practical advantages in public health systems where access to newer nephroprotective drugs remains limited.

Our study has several limitations. The most significant derives from sample size. Even though the calculated sample size met the required number of patients based on the expected effect on albuminuria, and the post hoc power estimate for LVMI was sufficient, the dropouts and the use of a per-protocol analytical strategy may have magnified the observed effect. It is essential to note that the original protocol anticipated a larger sample size and a longer follow-up period to address broader clinical objectives. Recruitment began shortly before the regional increase in COVID-19 cases, and enrollment was subsequently halted when the hospital was reassigned exclusively to COVID-19 care. Even so, the data are valuable, as they detected early positive changes after treatment initiation and showed parallel renal and cardiovascular effects, findings that have not been previously analyzed.

## 5. Conclusions

Pentoxifylline may be a safe and effective treatment with combined beneficial effects on kidney and heart in advanced diabetic nephropathy. However, results are exploratory and large RCTs with adequately power for hard outcomes such as ESRD, MACE, and death (cardiovascular and all-cause) and longer follow-up are needed particularly in settings where therapeutic alternatives are limited.

## Figures and Tables

**Table 1:** Baseline demographic, clinical, biochemical, and echocardiographic characteristics of the study participants.

	Placebo Group n = 41			Pentoxifylline Group n = 52			
	Mean		SD	Mean		SD	*p*
Sex (% male)	48.80			59.60			0.401
AGE (Years)	61.39	±	8.20	59.88	±	7.26	0.351
Systolic blood pressure 24 h (mmHg)	137.85	±	15.92	137.50	±	18.05	0.922
Diastolic blood pressure 24 h (mmHg)	73.68	±	7.51	73.54	±	8.41	0.932
Body weight (kg)	73.24	±	14.72	77.23	±	18.02	0.254
Body Mass Index (Kg/m^2^)	29.58	±	5.05	30.11	±	6.79	0.681
Waist/Hip circumference Index	0.98	±	0.06	0.99	±	0.07	0.481
Hemoglobin (g/dL)	13.31	±	1.60	13.25	±	1.85	0.856
Hematocrit (%)	39.01	±	4.32	39.20	±	5.04	0.844
Serum Glucose (mg/dL)	134.50	±	47.63	127.29	±	44.44	0.453
Glycosylated hemoglobin (%)	8.70	±	2.68	8.85	±	2.36	0.767
Serum Creatinine (mg/dL)	1.70	±	0.66	1.91	±	0.60	0.119
Cystatin C (mg/dL)	1.82	±	0.63	1.99	±	0.67	0.216
Urea (mg/dL)	63.90	±	23.80	66.49	±	22.21	0.590
Total serum Cholesterol (mg/dL)	185.15	±	50.29	180.62	±	47.91	0.659
HDL Cholesterol (mg/dL)	43.77	±	14.36	43.77	±	11.31	0.999
LDL Cholesterol (mg/dL)	113.42	±	47.43	113.41	±	48.17	0.999
Triglycerides (mg/dL)	205.56	±	109.51	208.23	±	138.31	0.920
Serum Albumin (g/dL)	4.02	±	0.56	3.89	±	0.40	0.204
Total serum Protein (g/dL)	7.26	±	0.81	7.22	±	0.74	0.815
C-Reactive Protein (mg/dL)	8.41	±	12.78	6.22	±	2.06	0.227
Serum Calcium (mg/dL)	9.45	±	0.61	9.49	±	0.64	0.727
Serum Phosphorus (mg/dL)	4.35	±	0.59	4.44	±	0.71	0.541
Serum Alkaline Phosphatase	113.44	±	50.62	109.40	±	39.74	0.668
Sodium (mEq/L)	138.71	±	2.32	138.98	±	4.24	0.711
Potassium (mEq/L)	4.87	±	0.51	4.74	±	0.49	0.200
Chlorine (mEq/L)	103.80	±	4.21	104.37	±	4.95	0.564
NT_PRO_BNP (pg/mL)	2137.58	±	1019.46	1894.89	±	631.73	0.235
Total body water (% BW)	51.25	±	6.74	52.52	±	7.37	0.393
Extracellular water (%BW)_PR_6m	23.28	±	2.22	23.70	±	3.05	0.466
Intracellular water (%BW)	28.08	±	3.61	29.02	±	3.91	0.235
Urine Volume (mL/24 h)	2078.44	±	726.51	2121.23	±	834.74	0.796
Urine protein (mg/dL)	149.20	±	259.70	166.29	±	193.95	0.717
Microalbuminuria (mg/24 h)	75.90	±	73.81	109.13	±	90.89	0.061
Creatinine clearance (mL/min/1.73 m^2^)	44.71	±	19.21	38.82	±	16.91	0.119
Left Ventricle mass (g)	195.86	±	56.31	198.00	±	46.72	0.850
Left Ventricle Mass Index (g/m^2^)	108.30	±	30.69	111.33	±	22.25	0.602
Left Ventricle Ejection Fraction (%)	67.37	±	8.86	67.22	±	7.77	0.937

**Table 2:** Changes in Clinical, Biochemical, and Body Composition Parameters from Baseline to 6 and 12 Months in the Pentoxifylline and Control Groups.

Variable	∆ from Baseline to 6 Months							∆ from Baseline to 12 Months						
	P-G			PTF-G				P-G			PTF-G			
	Mean	±	SD	Mean	±	SD	*p*	Mean	±	SD	Mean	±	SD	*p*
Weight (Kg)	−0.54	±	4.39	−0.97	±	5.85	0.695	−0.54	±	4.39	−0.97	±	5.85	0.580
Systolic blood pressure (mmHg)	−0.85	±	12.89	−1.42	±	13.33	0.841	−2.95	±	12.36	−1.04	±	16.12	0.651
Diastolic blood pressure (mmHg)	−2.00	±	6.88	−1.15	±	6.15	0.543	−2.55	±	8.82	−2.08	±	7.31	0.844
TBW (%BW)	−0.54	±	4.39	−0.97	±	5.85	0.923	2.73	±	6.45	0.35	±	3.96	0.130
ECW (%BW)	2.73	±	6.45	0.35	±	3.96	0.808	2.73	±	6.45	0.35	±	3.96	0.048
ICW (%BW)	0.18	±	0.92	0.02	±	2.20	0.665	0.42	±	1.00	0.10	±	1.46	0.393
Body fat (%BW)	−0.51	±	0.25	−0.58	±	4.03	0.926	−0.75	±	3.28	0.63	±	4.25	0.216
sGlucose (mg/dL)	−6.80	±	56.11	8.98	±	81.42	0.293	−18.31	±	71.00	−8.63	±	61.40	0.587
Glycated hemoglobin (%)	2.73	±	6.45	0.35	±	3.96	0.980	2.73	±	6.45	0.35	±	3.96	0.623
sCholesterol (mg/dL)	−14.22	±	40.90	−6.36	±	45.10	0.391	−8.96	±	38.22	−11.50	±	47.48	0.832
Triglycerides (mg/dL)	−21.07	±	110.74	−12.26	±	113.44	0.710	−16.67	±	96.19	−17.50	±	89.57	0.974
sAlbumin (g/dL)	−0.03	±	0.39	−0.04	±	0.37	0.878	−0.20	±	0.45	−0.07	±	0.45	0.330
ESR (%)	−0.07	±	11.04	−6.02	±	9.78	0.007	−5.78	±	12.31	−5.37	±	14.63	0.915
C-Reactive protein (mg/dL)	−0.06	±	4.36	−0.79	±	2.97	0.731	−0.74	±	2.80	−0.11	±	4.30	0.561

Data are presented as mean ± standard deviation. Comparisons between groups were performed using Student’s *t*-test. Significant *p* values indicate differences in the change from baseline between the Pentoxifylline and Control groups at each time point. TBW: total body water; ECW: extracellular water; ICW: intracellular water; ESR: erythrocyte sedimentation rate.

## Data Availability

The data presented in this study are available on reasonable request from the corresponding author. The data are not publicly available due to institutional policies and ethical restrictions associated with clinical trial participants.

## Abbreviations

The following abbreviations are used in this manuscript:

PTF	Pentoxifylline
P-G	Placebo Group
PTF-G	Pentoxifylline Group
DM2/T2DM	Type 2 Diabetes Mellitus
CKD	Chronic Kidney Disease
DN	Diabetic Nephropathy
eGFR	Estimated Glomerular Filtration Rate
eGFRCysCCr	Creatinine-Cystatin C-based Estimated GFR
LVMI	Left Ventricular Mass Index
NT-proBNP	N-terminal pro-Brain Natriuretic Peptide
KDIGO	Kidney Disease: Improving Global Outcomes
RAAS	Renin–Angiotensin–Aldosterone System
ACEI	Angiotensin-Converting Enzyme Inhibitor
ARB	Angiotensin II Receptor Blocker
SEM	Standard Error of the Mean
SGLT2i	Sodium-Glucose Cotransporter-2 Inhibitors
CRF	Case Report Form
IMSS	Instituto Mexicano del Seguro Social
APC	Article Processing Charge
IRB	Institutional Review Board
CC BY	Creative Commons Attribution License

## References

[1] Johnston-Webber C., Bencomo-Bermudez I., Wharton G., van Kessel R., Barone S., Muntó F.B., Chadban S., Sanchez J.J.G., Kocks J.W.H., Obolensky K. (2025). A conceptual framework to assess the health, socioeconomic and environmental burden of chronic kidney disease. Health Policy.

[2] Ogle G.D., Wang F., Haynes A., Gregory G.A., King T.W., Deng K., Dabelea D., James S., Jenkins A.J., Li X. (2025). Global type 1 diabetes prevalence, incidence, and mortality estimates 2025: Results from the International diabetes Federation Atlas, 11th Edition, and the T1D Index Version 3.0. Diabetes Res. Clin. Pr..

[3] (2024). Kidney Disease: Improving Global Outcomes (KDIGO) CKD Work Group. KDIGO 2024 Clinical Practice Guideline for the Evaluation and Management of Chronic Kidney Disease. Kidney Int..

[4] Ward A., Clissold S.P. (1987). Pentoxifylline. A review of its pharmacodynamic and pharmacokinetic properties, and its therapeutic efficacy. Drugs.

[5] Solerte S.B., Fioravanti M., Bozzetti A., Schifino N., Patti A.L., Fedele P., Viola C., Ferrari E. (1986). Pentoxifylline, albumin excretion rate and proteinuria in type I and type II diabetic patients with microproteinuria. Results of a short-term randomized study. Acta Diabetol. Lat..

[6] Guerrero-Romero F., Rodríguez-Morán M., Paniagua-Sierra J.R., García-Bulnes G., Salas-Ramírez M., Amato D. (1995). Pentoxifylline reduces proteinuria in insulin-dependent and non-insulin-dependent diabetic patients. Clin. Nephrol..

[7] Navarro-González J.F., Mora-Fernández C., de Fuentes M.M., Chahin J., Méndez M.L., Gallego E., Macía M., del Castillo N., Rivero A., Getino M.A. (2015). Effect of pentoxifylline on renal function and urinary albumin excretion in patients with diabetic kidney disease: The PREDIAN trial. J. Am. Soc. Nephrol..

[8] Gallardo J.M., Prado-Uribe M.C., Amato D., Paniagua R. (2007). Inflammation and oxidative stress markers by pentoxifillyne treatment in rats with chronic renal failure and high sodium intake. Arch. Med. Res..

[9] Navarro-González J.F., Muros M., Mora-Fernández C., Herrera H., Meneses B., García J. (2011). Pentoxifylline for Renoprotection in Diabetic Nephropathy: The PREDIAN study. Rationale and basal results. J. Diabetes Complicat..

[10] González-Espinoza L., Rojas-Campos E., Medina-Pérez M., Peña-Quintero M., Gómez-Navarro B., Cueto-Manzano A.M. (2012). Pentoxifylline decreases serum levels of tumor necrosis factor alpha, interleukin 6 and C-reactive protein in hemodialysis patients: Results of a randomized double-blind, controlled clinical trial. Nephrol. Dial. Transpl..

[11] McCormick B.B., Sydor A., Akbari A., Fergusson D., Doucette S., Knoll G. (2008). The effect of pentoxifylline on proteinuria in diabetic kidney disease: A meta-analysis. Am. J. Kidney Dis..

[12] Shan D., Wu H.M., Yuan Q.Y., Li J., Zhou R.L., Liu G.J. (2012). Pentoxifylline for diabetic kidney disease. Cochrane Database Syst. Rev..

[13] Tian M.L., Shen Y., Sun Z.L., Zha Y. (2015). Efficacy and safety of combining pentoxifylline with angiotensin-converting enzyme inhibitor or angiotensin II receptor blocker in diabetic nephropathy: A meta-analysis. Int. Urol. Nephrol..

[14] Leporini C., Pisano A., Russo E., D Arrigo G., de Sarro G., Coppolino G., Bolignano D. (2016). Effect of pentoxifylline on renal outcomes in chronic kidney disease patients: A systematic review and meta-analysis. Pharmacol. Res..

[15] Jiang X., Zhou S., Yao J., Kong X., Cui M. (2016). Effect of pentoxifylline in proteinuric chronic kidney disease: A systematic review and meta-analysis. J. Nephrol..

[16] Liu D., Wang L.N., Li H.X., Huang P., Qu L.B., Chen F.Y. (2017). Pentoxifylline plus ACEIs/ARBs for proteinuria and kidney function in chronic kidney disease: A meta-analysis. J. Int. Med. Res..

[17] Zhang M., Wang Y., Fu W., Sun L. (2024). The effect of a methylxanthine vasodilator: Pentoxifylline on the treatment of diabetic nephropathy-a meta-analysis. Naunyn Schmiedebergs Arch. Pharmacol..

[18] Donate-Correa J., Ferri C.M., Mora-Fernández C., Pérez-Delgado N., González-Luis A., Navarro-González J.F. (2024). Pentoxifylline ameliorates subclinical atherosclerosis progression in patients with type 2 diabetes and chronic kidney disease: A randomized pilot trial. Cardiovasc. Diabetol..

[19] Li R., Zhang X., Xu Y., Feng T. (2024). Vascular endothelial dysfunction improvements in patients with uremia using pentoxifylline-suppressing nlrp3 expressions and hmgb1 release. Shock.

[20] Yang Q., Lang Y., Yang W., Yang F., Yang J., Wu Y., Xiao X., Qin C., Zou Y., Zhao Y. (2023). Efficacy and safety of drugs for people with type 2 diabetes mellitus and chronic kidney disease on kidney and cardiovascular outcomes: A systematic review and network meta-analysis of randomized controlled trials. Diabetes Res. Clin. Pr..

[21] Lang R.M., Badano L.P., Mor-Avi V., Afilalo J., Armstrong A., Ernande L., Flachskampf F.A., Foster E., Goldstein S.A., Kuznetsova T. (2015). Recommendations for cardiac chamber quantification by echocardiography in adults: An update from the American Society of Echocardiography and the European Association of Cardiovascular Imaging. J. Am. Soc. Echocardiogr..

[22] Devereux R.B., Alonso D.R., Lutas E.M., Gottlieb G.J., Campo E., Sachs I., Reichek N. (1986). Echocardiographic assessment of left ventricular hypertrophy: Comparison to necropsy findings. Am. J. Cardiol..

[23] Mosteller R.D. (1987). Simplified calculation of body-surface area. N. Engl. J. Med..

[24] Nagueh S.F., Smiseth O.A., Appleton C.P., Byrd B.F., Dokainish H., Edvardsen T., Flachskampf F.A., Gillebert T.C., Klein A.L., Lancellotti P. (2016). Recommendations for the Evaluation of Left Ventricular Diastolic Function by Echocardiography: An Update from the American Society of Echocardiography and the European Association of Cardiovascular Imaging. J. Am. Soc. Echocardiogr..

[25] Hintze J. (2008). PASS 2008.

[26] Biju B.K., Andavar M. (2025). Exploring the role of pentoxifylline as a renal protector in diabetic kidney disease: A comprehensive review. BMC Nephrol.

[27] Champion S., Lapidus N., Cherié G., Spagnoli V., Oliary J., Solal A.C. (2014). Pentoxifylline in heart failure: A meta-analysis of clinical trials. Cardiovasc. Ther..

[28] McCarty M.F., O’Keefe J.H., DiNicolantonio J.J. (2016). Pentoxifylline for vascular health: A brief review of the literature. Open Heart.

[29] Leehey D.J. (2020). Targeting Inflammation in Diabetic Kidney Disease: Is There a Role for Pentoxifylline?. Kidney360.

[30] de Morales A.M., Goicoechea M., Verde E., Carbayo J., Barbieri D., Delgado A., Verdalles U., de Jose A.P., Luño J. (2019). Pentoxifylline, progression of chronic kidney disease (CKD) and cardiovascular mortality: Long-term follow-up of a randomized clinical trial. J. Nephrol..

[31] Kuo K.L., Hung S.C., Liu J.S., Chang Y.K., Hsu C.C., Tarng D.C. (2015). Add-on Protective Effect of Pentoxifylline in Advanced Chronic Kidney Disease Treated with Renin-Angiotensin-Aldosterone System Blockade—A Nationwide Database Analysis. Sci. Rep..

[32] Chen P.M., Lai T.S., Chen P.Y., Lai C.F., Wu V., Chiang W.C., Chen Y.M., Wu K.D., Tsai T.J. (2014). Renoprotective effect of combining pentoxifylline with angiotensin-converting enzyme inhibitor or angiotensin II receptor blocker in advanced chronic kidney disease. J. Formos. Med. Assoc..

[33] Skudicky D., Bergemann A., Sliwa K., Candy G., Sareli P. (2001). Beneficial effects of pentoxifylline in patients with idiopathic dilated cardiomyopathy treated with angiotensin-converting enzyme inhibitors and carvedilol: Results of a randomized study. Circulation.

[34] Sliwa K., Woodiwiss A., Candy G., Badenhorst D., Libhaber C., Norton G., Skudicky D., Sareli P. (2002). Effects of pentoxifylline on cytokine profiles and left ventricular performance in patients with decompensated congestive heart failure secondary to idiopathic dilated cardiomyopathy. Am. J. Cardiol..

[35] Watanabe H., Furukawa Y., Chiba S. (1982). Cardiovascular effects of aminophylline and pentoxifylline on intact dogs and isolated dog atria. Jpn. Heart J..

[36] Zhang X., Meng F., Song J., Zhang L., Wang J., Li D., Li L., Dong P., Yang B., Chen Y. (2016). Pentoxifylline Ameliorates Cardiac Fibrosis, Pathological Hypertrophy, and Cardiac Dysfunction in Angiotensin II-induced Hypertensive Rats. J. Cardiovasc. Pharmacol..

[37] Brie D., Sahebkar A., Penson P.E., Dinca M., Ursoniu S., Serban M.C., Zanchetti A., Howard G., Ahmed A., Aronow W.S. (2016). Effects of pentoxifylline on inflammatory markers and blood pressure: A systematic review and meta-analysis of randomized controlled trials. J. Hypertens..

[38] Bell D.S.H., Jerkins T. (2024). The potential for improved outcomes in the prevention and therapy of diabetic kidney disease through ‘stacking’ of drugs from different classes. Diabetes Obes. Metab..

